# Asian elephants exhibit post-reproductive lifespans

**DOI:** 10.1186/s12862-019-1513-1

**Published:** 2019-10-21

**Authors:** Simon N. Chapman, John Jackson, Win Htut, Virpi Lummaa, Mirkka Lahdenperä

**Affiliations:** 1 Department of Biology, University of Turku, Turku, Finland; 2 Department of Animal and Plant Sciences, University of Sheffield, Sheffield, UK; 3 Myanma Timber Enterprise, Ministry of Natural Resources and Environment Conservation, Yangon, Myanmar

**Keywords:** Asian elephant, Demography, Fertility, Long-term data, Post-reproductive life, Reproductive cessation

## Abstract

**Background:**

The existence of extended post-reproductive lifespan is an evolutionary puzzle, and its taxonomic prevalence is debated. One way of measuring post-reproductive life is with post-reproductive representation, the proportion of adult years lived by females after cessation of reproduction. Analyses of post-reproductive representation in mammals have claimed that only humans and some toothed whale species exhibit extended post-reproductive life, but there are suggestions of a post-reproductive stage for false killer whales and Asian elephants. Here, we investigate the presence of post-reproductive lifespan in Asian elephants using an extended demographic dataset collected from semi-captive timber elephants in Myanmar. Furthermore, we investigate the sensitivity of post-reproductive representation values to availability of long-term data over 50 years.

**Results:**

We find support for the presence of an extended post-reproductive stage in Asian elephants, and that post-reproductive representation and its underlying demographic rates depend on the length of study period in a long-lived animal.

**Conclusions:**

The extended post-reproductive lifespan is unlikely due to physiological reproductive cessation, and may instead be driven by mating preferences or condition-dependent fertility. Our results also show that it is crucial to revisit such population measures in long-lived species as more data is collected, and if the typical lifespan of the species exceeds the initial study period.

## Background

The evolution of extended post-reproductive lifespan is a long-term puzzle in evolutionary biology, where extended refers to post-reproductive lifespan that is not an artefact of individual variation in somatic and reproductive senescence rates [1]. Species with extended post-reproductive lifespans are implicitly considered to be physiologically incapable of further reproduction, and though post-reproductive lifespan is often considered rare [2, 3], others claim it to be a general mammalian trait [4–6]. This controversy is due to a combination of methodological differences (reviewed in [7]), variation in definitions of what constitutes a post-reproductive period, and the fact that post-reproductive lifespan is mathematically constrained to be positive unless all females immediately die after reproduction. An age-specific decline in fertility is common among animals [8, 9], and iteroparous animals are generally expected to exhibit declining fertility as a function of overall senescence [10]. However, even female primates, our nearest relatives, retain fertility close to the end of their lives [11, 12], resulting in a maximum post-reproductive lifespan of a few years in these species [4]. Therefore, whilst some degree of post-reproductive lifespan is common across animals, post-reproductive lifespans extending well beyond regular birth intervals and covering several years or even decades are rare across the animal kingdom.

To distinguish extended post-reproductive lifespan from individual variation in senescence, measures that do not depend on any one individual are preferable to those calculated off specific individuals (e.g. expected lifespan vs maximum recorded lifespan) or individuals meeting specific criteria [7, 13]. Individual-based measures are often correlated with overall longevity and may introduce biases through exclusion of individuals considered to have died before being able to reproduce again (see [7] for further discussion of these issues). Post-reproductive representation (PrR) is a population measure that does not depend on specific individuals or limiting criteria [7], and is defined as the proportion of total adult lifespan spent in a post-reproductive state. PrR is calculated by dividing the number of years an average female newborn is expected to live post-reproductively by the total number of years she is expected to live as an adult [7, 13]. Though the applicability of PrR to wild populations has been questioned due to the difficulty in obtaining sufficient data [6], recent work has not only shown that PrR can be measured for wild populations, but that it remains statistically robust even in the absence of long-term data [3, 14], assuming that estimated ages are accurate and a representative cross-section of a population is used. However, these conditions may not always be fulfilled in longitudinal datasets, especially if the lifespan of the animal greatly exceeds the length of the study period. Furthermore, full captivity is problematic for assessing post-reproductive life: reproductive senescence can be accelerated by breeding programmes in zoos, and exclusion of extrinsic causes of mortality can extend average lifespan [1]. Therefore it is important to assess the robustness of the dataset and to carefully select the population when estimating the existence of post-reproductive life in a species, and to use appropriate methods to overcome the acknowledged limitations of the data.

Whilst humans display a distinct and obvious end to reproduction - menopause - they are not the only species with an extended post-reproductive lifespan. It is currently thought that a ‘true’ post-reproductive lifespan, with a substantial proportion of females in the population being post-reproductive and living notably long thereafter, is a trait exhibited by a limited number of mammalian species (which are also physiologically incapable of further reproduction): humans, killer whales (*Orcinus orca*), narwhals (*Monodon monoceros*), beluga whales (*Delphinapterus leucas*), and short-finned pilot whales (*Globicephala macrorhynchus*) [3, 14]. All these species exhibit a PrR or physiological-PrR value (see [14]) between 0.15 and 0.30 i.e. 15–30% of adult female years are lived by post-reproductive individuals. False killer whales (*Pseudorca crassidens*) may also exhibit some degree of extended post-reproductive life (PrR = 0.14 [15], but see also [14]).

The PrR analyses of Ellis et al. [3] covered 52 mammal species with wild populations for which life tables could be constructed. However, this did not include every mammal species that could potentially exhibit extended post-reproductive life - those with similar life-history traits, including social structure, to species with extended post-reproductive life. For example, whilst African elephants (*Loxodonta africana*) were included in this study and were reported to have a low PrR of 0.04 (i.e. not post-reproductive), Asian elephants (*Elephas maximus*) were not analysed. Asian elephants have actually been shown to have a PrR shorter than that of ‘true’ post-reproductive species, but much longer than that of non-post-reproductive mammals (PrR = 0.13 [16]), although the significance of this has not been tested. Despite broad similarities in social structure and lifespan, African and Asian elephants are markedly different species, with mitochondrial DNA suggesting that divergence occurred 5.43–8.42 million years ago (reviewed in [17]). Ecologically, Asian elephants are distinct from African elephants, having smaller body and group sizes, and occupying the forest-grassland ecotone [18]. The fact that Asian elephants are long-lived and occur in forested habitat has made a detailed understanding of demographic variation in wild populations all but impossible (but see [19]). However, semi-captive populations in range states have the potential to provide insights into the life-history traits of this enigmatic long-lived species. Here, we first investigate the existence of extended post-reproductive lifespan and the significance of PrR in Asian elephants, with a larger, longer-term demographic dataset than that previously used by Lahdenperä et al. [16]. Second, though Ellis et al. [3] showed that the PrR method is robust for cross-sectional and most longitudinal data, they did not have available data to show whether the methodology is also robust for longitudinally-sampled populations of long-lived species. We therefore investigate how values of PrR and underlying/related demographic variables change depending on the length of study, from 1960 to 2018.

To address PrR in Asian elephants, and more generally how the length of the field study affects the outcome of PrR estimates, we use detailed demographic records of 3802 females from timber camps in the Union of Myanmar, all born within the last 80 years (approximately 3–4 generations). Despite individuals being held in captivity and used in the extraction of timber, they are more frequently described as semi-captive. Crucially for the analysis of post-reproductive lifespan, mortality [20] and fertility [21] patterns in this population have been compared to wild populations, including African elephants, and distinguished from captive populations held in zoos, which have much lower survival and fertility rates. Furthermore, individuals are not culled, receive only basic veterinary care, and are not subjected to reproductive management [22]. Thus, we believe that the current study population is appropriate for the study of PrR in Asian elephants, and a valuable resource for understanding the rare occurrence of post-reproductive lifespan in mammals. More generally, a longitudinal dataset of an exceptionally long-lived mammal offers the opportunity to assess how sensitive the PrR estimation method is to truncated life histories and the instability of population structure through time (see [23]).

## Methods

### Study population

Asian elephants are listed as endangered on the IUCN Red List of Threatened Species, but, unusually for an endangered species, have a large captive population of 16,000 individuals [24]. The largest captive population of elephants is in the Union of Myanmar, where ~ 2700 state-owned elephants are currently utilised in timber extraction, which are managed by the Myanma Timber Enterprise and monitored for the current study. We formally describe this population as semi-captive for several reasons. First, elephants are used in timber extraction between June and February and work during the day, but outside daily working hours, and at night, individuals are free-roaming and forage naturally (i.e. aside from the occasional seasonal fruit, or rice if travelling longer distances, there is no food supplementation). They also roam freely in the three-month annual rest period. Second, there is no reproductive management or husbandry in the population and individuals mate freely and receive no help with calving. Third, humans do not intervene with the care of calves, who receive maternal and allomaternal care until they are trained from the age of four [25, 26]. Finally, culling is not practised under any circumstance regardless of working ability, and elephants only have access to basic veterinary care. This care covers wound and abscess treatment, diagnosis and treatment of basic gastrointestinal diseases, vaccinations against anthrax and haemorrhagic septicaemia, and twice-yearly deworming. However, before 2000 (covering the majority of the data used here), modern veterinary care was minimal, and more than 40% of deaths in this population are directly attributable to acute or chronic illnesses or parasite infection [27].

Timber elephants are monitored by the Myanma Timber Enterprise and the current dataset has been compiled through individual logbooks and end-of-year reports (see e.g. [28] for further details). Demographic information including date of birth and origin (captive-born, for those born to a mother already in the population, or wild-caught, for those born in the wild and captured later on), capture date (if wild-caught), date of death or date last seen, and any calves is recorded for all registered elephants. Captive-born elephants have known dates of birth, and the age of wild-caught elephants has been estimated based on a number of measures [28, 29].

We included all captive-born females born after 1940 (reaching reproductive age in the 1950s, when record-keeping was more consistent), and any wild-caught females entering the population after 1951. As age-estimates for wild-caught individuals may have a lower accuracy after full body height is achieved [30], we included only wild-caught females captured before the age of 25. Finally, individuals with erroneous or discontinuous birth/entry and death/departure information were also removed (~ 5% of elephants). The final dataset contained demographic information for 3802 females from 1940 until 2018.

### Constructing life tables

We constructed life tables for females from longitudinal censored data, using R v3.5.1 [31]. Individuals were followed until death, unless they were last recorded as still alive in the log books, in which case they were censored at the time of last recording.

The total number of individuals at age *x* (in years) were known, and from this we derived probabilities of survival to each age (*l*_*x*_). This was done for all elephants in the sample (*n* = 3802), but also for only captive-born elephants (*n* = 2568). We obtained *l*_*x*_ from Cox proportional hazards models, which account for censoring. These were implemented with the *Surv* and *survfit* functions from the *survival* package [32]. For the *l*_*x*_ series – the ordered sequence of age-specific *l* values - in which elephants caught from the wild were included, we created a left-censored *Surv* object in R, with time set as the estimated age at capture for wild-caught individuals, and as 0 years for captive-born individuals. The second time argument was then set as 1 + age at death or 1 + age of censoring, as the models do not accept identical entry and exit ages (e.g. elephants dying in the year of birth or capture). This one-year shift was corrected for after analysis by removing the value for age 0 (which always showed all elephants surviving).

For these two datasets, we also modified the *l*_*x*_ series by decreasing the maximum age by 1 and 5 years, to see how shortening lifespan would affect PrR. This was effectively a proxy of a hypothetical wild population, under the assumption that the semi-captive elephants are living prolonged life because of their partial captivity. However, we again wish to emphasise that Asian elephants actually live shorter lives in zoos than in the working population of Myanmar [20], and therefore these *l*_*x*_-altered populations may be closer to the situation for zoo Asian elephants rather than wild. For the 1-year reductions, we replaced the last value of *l*_*x*_ with 0. For the 5-year reduction, *l*_*x*_ was modified by removing the ‘extreme’ ages, which involved replacing the last 5 values at the end of the *l*_*x*_ series with 0 s. Due to fairly low sample sizes at older ages, this did not remove too many observations of long-lived elephants (*l*_*x-1*_: 1 individual/1 observation; *l*_*x-5*_: 5 individuals/14 observations; captive only *l*_*x-1*_: 4 individual/4 observations; captive only *l*_*x-5*_: 15 individuals/39 observations).

We then calculated fecundity at each given age (*m*_*x*_). This was determined using the birth records from individual-based log books, and provides clear evidence of female reproductive activity. To obtain *m*_*x*_, we divided the number of offspring of either sex born in a year by *L*_*x*_, the number of individual years lived between age_*x*_ and age_*x + 1*_ (number of individuals at age_*x*_ - half the number of individuals dying at age_*x*_). In addition to calculating the *m*_*x*_ series for the whole sample, we also calculated it for a subset containing captive-born elephants only.

Though the age at first reproduction for wild Asian elephants is currently unclear, zoo elephants have an earlier and shorter reproductive period than this semi-captive elephant population [21]. The reproduction of wild Asian elephants is probably not dissimilar to this population: first, the elephants in this population reproduce without human intervention (e.g. artificial insemination), and second, from what little demographic data is available for wild Asian elephants, no females in the wild which are thought to be over age 60 are known to have reproduced [19]. As such, we did not modify the *m*_*x*_ series.

### Calculating post-reproductive life

After constructing the life tables to get the *l*_*x*_ and *m*_*x*_ series, we calculated PrR from each series. To mathematically describe PrR, we must first define additional demographic notation. In addition to *l*_*x*_ (the proportion of individuals surviving to age_*x*_), PrR requires calculation of *e*_*x*_ (life expectancy at age_*x*_). Multiplication of these measures provides *T*_*x*_, the total individual years lived after *x*. PrR can then be calculated from *T*_*x*_ at the ages of 5 and 95% population fecundity (age *B* and age *M* respectively) [7]. Age *B* and age *M* represent the ages at the beginning of adulthood and the end of fecundity. We quantified the PrR according to the following formula:

$$ PrR=\frac{T_M}{T_B}=\frac{l_M}{l_B}\ast \frac{e_M}{e_B} $$

We used existing R code from the literature to calculate ages *B* and *M*, and PrR for each sample (see ‘S3_Rcode’ from [7]). We applied a slight modification to this code to calculate the statistical significance of the PrR value, following the method used by Ellis et al. [3], by increasing the number of simulated populations to 9999 [33]. The *p* values for each PrR are calculated by dividing the number of simulated populations with a PrR exceeding the actual PrR by the number of simulations plus one (i.e. 10,000); see eq. 1 of Ruxton and Neuhäuser [33].

### Sensitivity of PrR to length of study

To investigate how the PrR value and related, relevant demographic rates are affected by data availability, we re-ran the analysis on a year-by-year basis from 1960 to 2018. To do this, we only considered known individuals and birth/death/censoring events in the population in each focal year. In effect, this mimics the situation if the study ended in 1960, 1961, 1962 etc. As above, only individuals born in captivity after 1940 or captured from the wild after 1951 were included. From this, the final dataset consisted of 59 *l*_*x*_ and *m*_*x*_ series. The code of Levitis and Lackey [7] was then used to calculate PrR, ages *B* and *M*, and *e*_*x*_ for the population on a yearly basis.

## Results

**Fig. 1:**
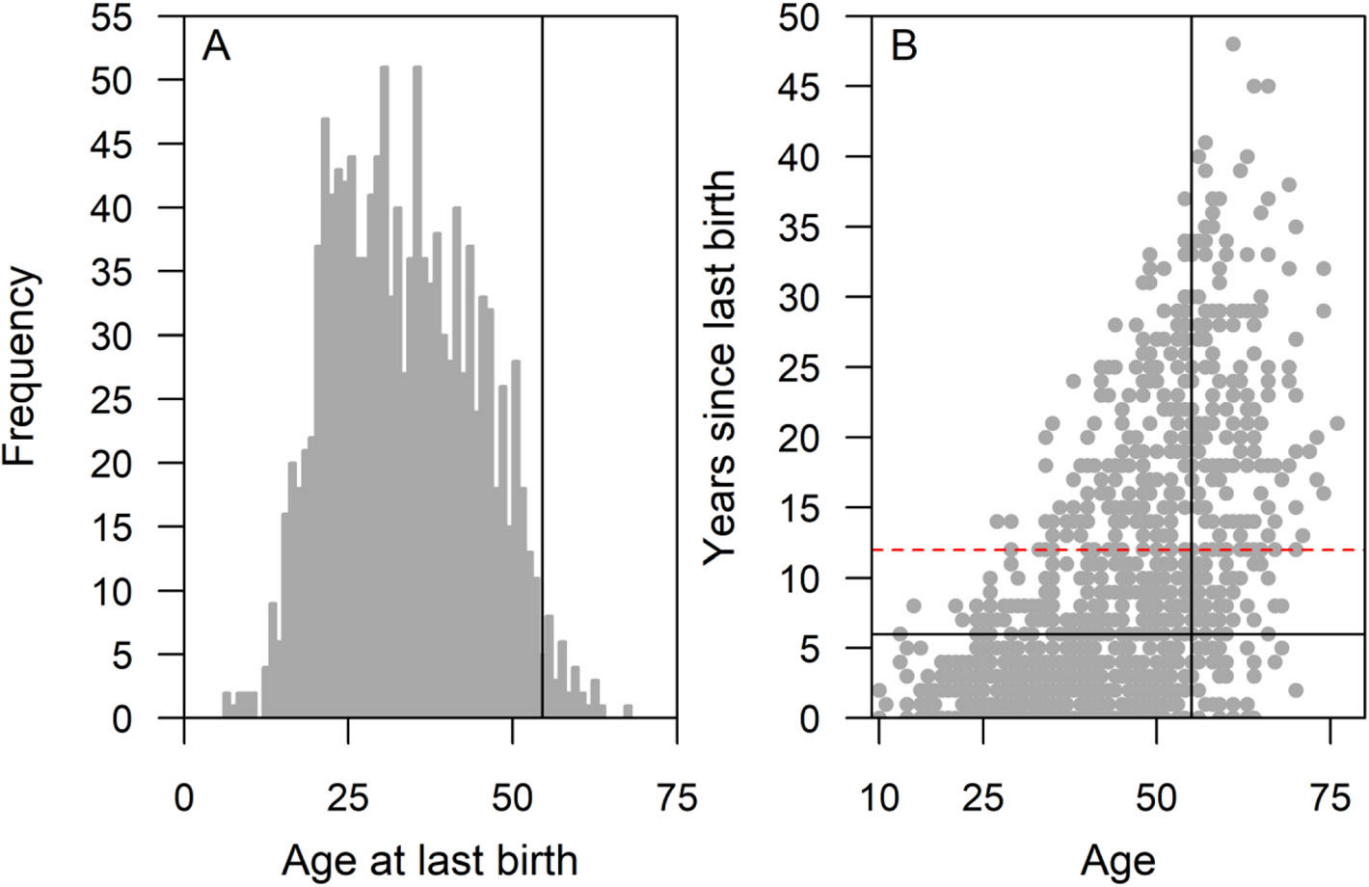
Age at latest birth in the Myanmar timber elephant population. Only females reproducing at least once are shown (*n* = 1298). Vertical lines indicate age at 95% population fecundity. **a** histogram of frequency of ages at last birth; **b** scatterplot of years since last birth by (current/death) age of the elephants. Horizontal lines show the mean inter-birth intervals (in black) and mean plus two standard deviations (dashed red), from [16]

The oldest elephants in our sample were 69 (captive-born) and 76 (wild-caught), and the oldest reproductive elephants were 55 (captive-born) and 64 (wild-caught); these reproductive ages are similar to the last known age at reproduction for a population of wild Asian elephants with estimated ages [19]. The distribution of the age at last (i.e. most recent) birth in the current population (Fig. 1a) shows that there is not a clearly defined population-level cessation in reproduction, as one might expect were the elephants to undergo physiological reproductive cessation. However, Fig. 1b provides an indication of the variation between individuals in the length of post-reproductive lifespan. There are a large number of older elephants - indicated by being of age *M* (age at 95% population fecundity; 55 years) and over - who have not reproduced for more than the mean (plus two standard deviations) inter-birth interval [16] and are thus potentially in a post-reproductive stage (*n* = 177; 65.6% of female elephants aged 55 or over).

### Post-reproductive representation

The post-reproductive representation of Asian elephants was significantly larger than zero (Table 1) in all cases, ranging between 0.148 for the *l*_*x-5*_ modified full population and 0.207 for the unmodified, captive-born only elephant population. That our modified populations were still similar shows the PrR method is, for this population at least, robust to the removal of the ‘extreme’ ages of the longest-lived elephants. We found a slight difference between the values for captive-born only (PrR = 0.207) and the full population (wild-caught and captive-born; PrR = 0.162).

**Table 1:** Post-reproductive representation (PrR) of Myanmar timber elephants

Origin	Modification	Age *B*	Age *M*	*e*_*B*_	*e*_*M*_	PrR	*p*
CB & WC	Base	17	55	25	5	0.162	< 0.001
	*l*_*x*_ *− 1*	17	55	25	5	0.159	< 0.001
	*l*_*x*_ *− 5*	17	55	25	5	0.148	< 0.001
CB	Base	17	50	20	7	0.207	< 0.001
	*l*_*x*_ *− 1*	17	50	20	7	0.205	< 0.001
	*l*_*x*_ *− 5*	17	50	20	7	0.192	< 0.001

PrR is a measure of the proportion of adult female years lived post-reproductively. CB refers to captive-born elephants, WC to wild-caught. Ages *B* and *M* are the ages at 5 and 95% population fecundity, respectively, whilst *e*_*B*_ and *e*_*M*_ are the expected female lifespan at ages *B* and *M* (rounded to the nearest whole number)

**Fig. 2:**
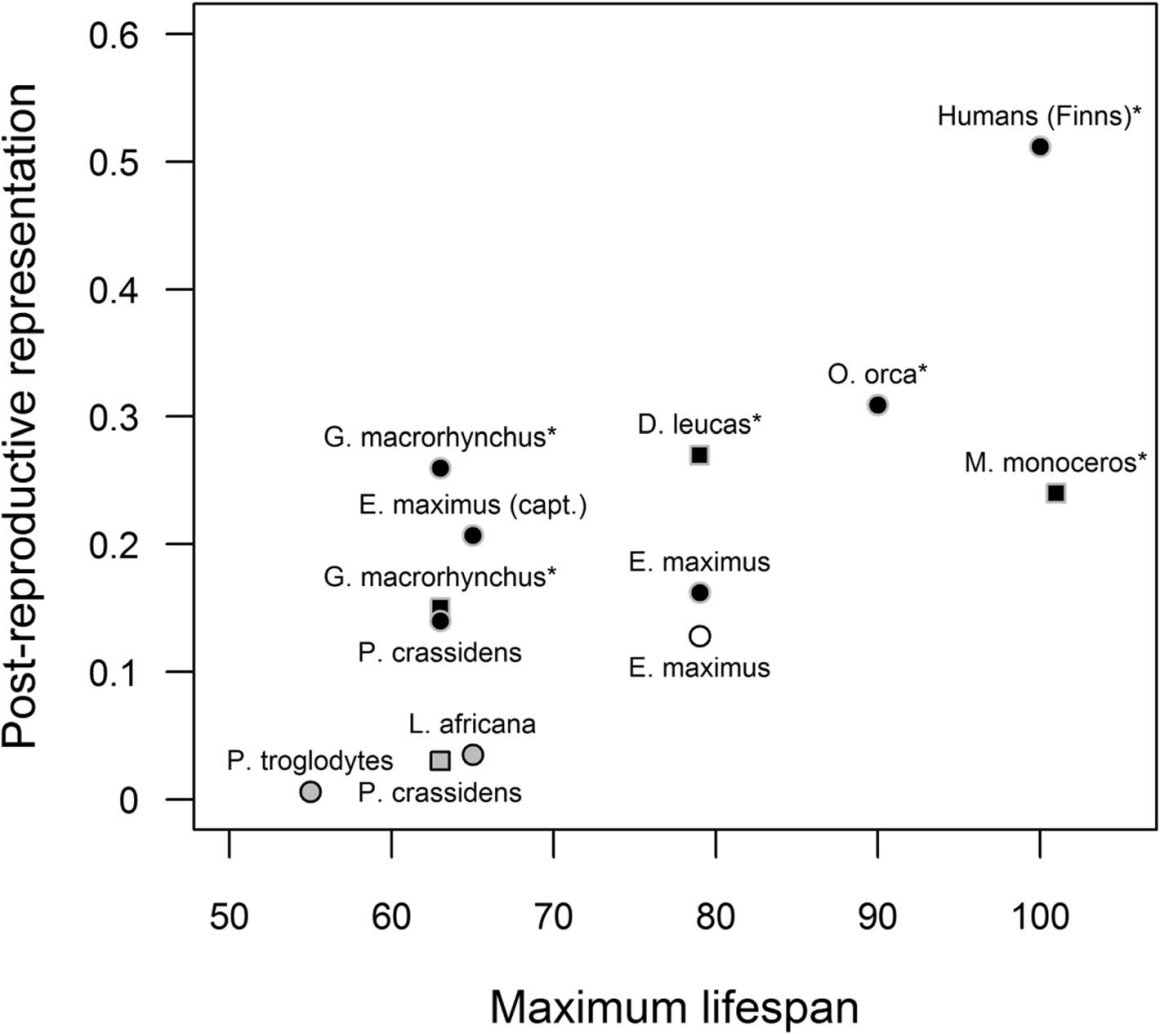
Postreproductive representation values of long-lived mammals by maximum female lifespan. Species labels are above or below their point, with * indicating the species has early reproductive cessation. Colour of points indicates significance of PrR- (circle; see [7]) and physiologically-derived PrR-values (square; see [14]): black for significant, grey for non-significant, and white for not assessed. PrR-values for humans and Asian elephants (*E. maximus*, white) from [16], Asian elephants (black) from this paper, killer whales (*O. orca*), chimpanzees (*P. troglodytes*), short-finned pilot whales (*G. macrorhychus*), and African elephants (*L. Africana*) from [3], false killer whales (*P. crassidens*) from [15], and physiological PrR-values for narwhals (*M. monoceros*), beluga whales (*D. leucas*), false killer whales, and short-finned pilot whales from [14]. Maximum female lifespan from [16] (Asian elephants and pre-industrial Finns), [34] (African elephants), [35] (killer whales), [15] (false killer whales), [36] (chimpanzees), [37] (short-finned pilot whales), [38] (narwhals), and [39] (beluga whales)

These PrR values are within the range of values for short-finned pilot whales, with the lowest value here (PrR = 0.148) slightly higher than the value for short-finned pilot whales with simulated population decline (PrR = 0.131) [3]. Whilst lower than most values of species known to have an early physiological end to reproductive ability (Fig. 2), the PrR observed here is still much larger than any species without early reproductive cessation, with the nearest PrR value coming from the yellow baboon *Papio cynocephalus* at 0.036 [3].

### Sensitivity of PrR to length of study

**Fig. 3:**
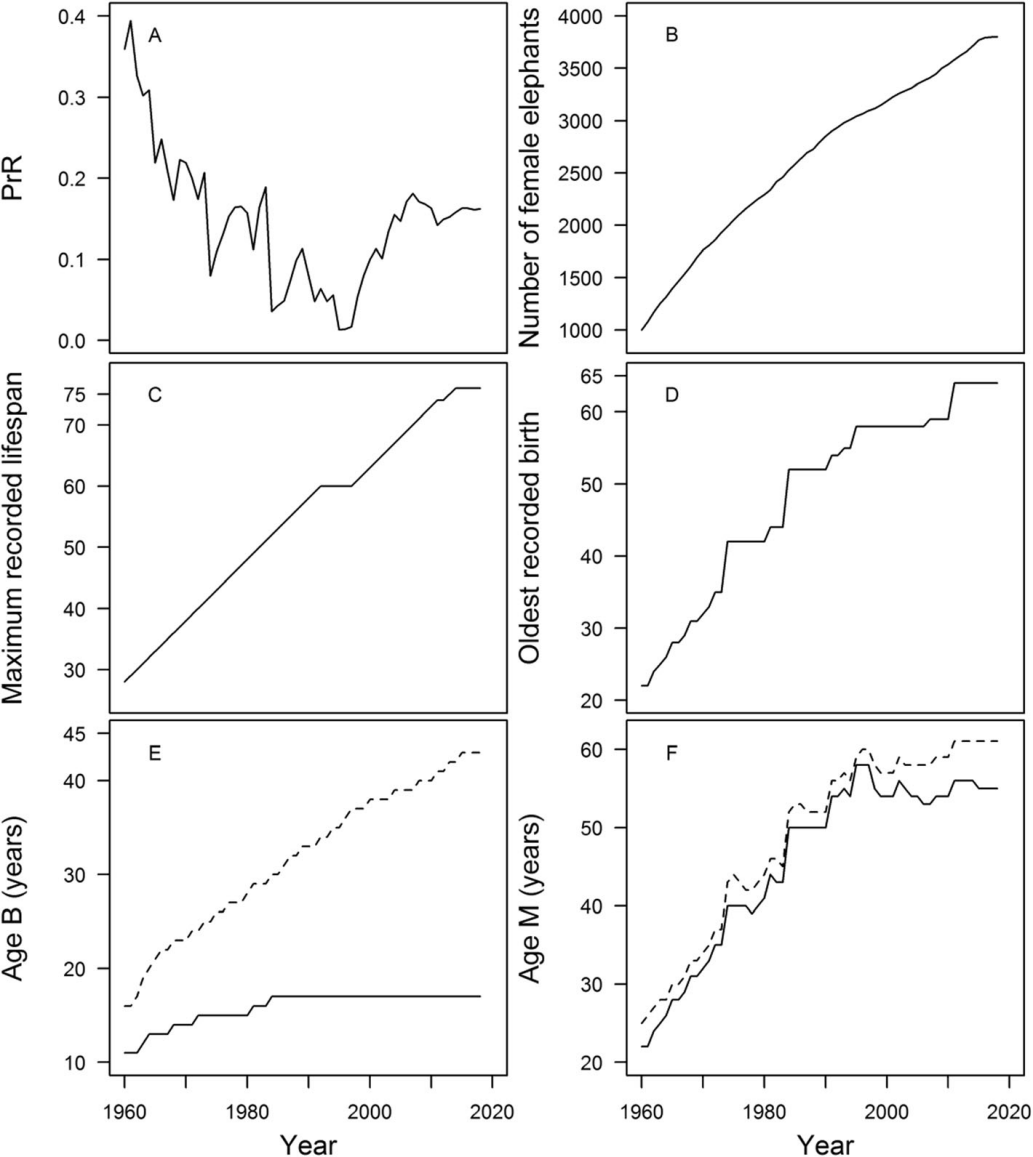
Changes in PrR and demographic values from 1960 to 2018. **a** post-reproductive representation; **b** number of individuals in the dataset; **c** maximum recorded lifespan; **d** oldest age at birth; **e** age at 5% population fecundity (age *B*; solid line) and expected lifespan for individuals reaching these ages (age *B* + *e*_*B*_; dashed line); **f** age at 95% population fecundity (age *M*; solid line) and expected lifespan for individuals reaching these ages (age *M* + *e*_*M*_; dashed line). See also Table 2

Our second aim was to quantify how values of PrR and its underlying demographic variables may change depending on the length of study. There was large variation in post-reproductive representation through time (Fig. 3a; Table 2), which was initially near the high values typical of humans (PrR > 0.3) [7], but declined over the study period. In the 1990s, the PrR values were very low (< 0.1), similar to those species described in Ellis et al. [3] as not having extended post-reproductive life. After 2000, the PrR value rose again, and appears to have begun to stabilise around 0.16 in recent years.

**Table 2:** Changes in PrR and demographic values through time

Cut-off year	N	PrR	Age *B*	Age *M*	*e*_*B*_	*e*_*M*_	Maximum lifespan	Oldest birth
1960	998	0.359	11	22	5	3	28	22
1961	1076	0.394	11	22	5	4	29	22
1962	1170	0.326	11	24	6	3	30	24
1963	1253	0.302	12	25	7	3	31	25
1964	1318	0.309	13	26	7	2	32	26
1965	1396	0.219	13	28	8	2	33	28
1966	1468	0.248	13	28	9	2	34	28
1967	1535	0.209	13	29	9	2	35	29
1968	1609	0.173	14	31	9	2	36	31
1969	1696	0.223	14	31	9	2	37	31
1970	1763	0.219	14	32	9	2	38	32
1971	1807	0.200	14	33	10	2	39	33
1972	1859	0.174	15	35	9	2	40	35
1973	1932	0.207	15	35	10	2	41	35
1974	1986	0.080	15	40	10	3	42	42
1975	2051	0.109	15	40	11	4	43	42
1976	2106	0.130	15	40	11	3	44	42
1977	2159	0.153	15	40	12	2	45	42
1978	2205	0.164	15	39	12	3	46	42
1979	2251	0.165	15	40	12	3	47	42
1980	2292	0.157	15	41	13	3	48	42
1981	2337	0.112	16	44	13	2	49	44
1982	2415	0.164	16	43	13	3	50	44
1983	2459	0.189	16	43	13	2	51	44
1984	2528	0.036	17	50	13	2	52	52
1985	2580	0.043	17	50	13	3	53	52
1986	2639	0.049	17	50	14	3	54	52
1987	2697	0.073	17	50	15	2	55	52
1988	2727	0.099	17	50	15	2	56	52
1989	2793	0.113	17	50	16	2	57	52
1990	2851	0.080	17	50	16	2	58	52
1991	2900	0.048	17	54	16	2	59	54
1992	2940	0.064	17	54	17	2	60	54
1993	2982	0.048	17	55	17	2	60	55
1994	3012	0.056	17	54	18	2	60	55
1995	3042	0.013	17	58	18	1	60	58
1996	3066	0.014	17	58	19	2	60	58
1997	3097	0.017	17	58	20	2	60	58
1998	3118	0.054	17	55	20	3	61	58
1999	3148	0.080	17	54	20	3	62	58
2000	3190	0.100	17	54	21	3	63	58
2001	3230	0.113	17	54	21	3	64	58
2002	3262	0.101	17	56	21	3	65	58
2003	3287	0.134	17	55	21	3	66	58
2004	3315	0.155	17	54	22	4	67	58
2005	3352	0.147	17	54	22	4	68	58
2006	3379	0.171	17	53	22	5	69	58
2007	3408	0.181	17	53	22	5	70	59
2008	3446	0.171	17	54	23	5	71	59
2009	3502	0.168	17	54	23	5	72	59
2010	3536	0.163	17	54	23	5	73	59
2011	3582	0.142	17	56	24	5	74	64
2012	3623	0.149	17	56	24	5	74	64
2013	3661	0.152	17	56	25	5	75	64
2014	3715	0.158	17	56	25	5	76	64
2015	3769	0.163	17	55	26	6	76	64
2016	3793	0.163	17	55	26	6	76	64
2017	3799	0.161	17	55	26	6	76	64
2018	3802	0.162	17	55	26	6	76	64

Ages *B* and *M* are the ages at 5 and 95% population fecundity, respectively, whilst *e*_*B*_ and *e*_*M*_ are the expected female lifespan at ages *B* and *M* (rounded to the nearest whole number)

One cannot consider PrR alone though, as its calculation relies heavily on other demographic variables. Whilst sample size is not directly incorporated into the PrR equation, it can have a large effect on the survival and fertility series. For example, one birth will have a greater impact on the fertility rate if the sample size at that age is smaller, which may be important for slow reproducers such as elephants. The same is true for the sampling of old individuals to determine, for example, the maximum lifespan or expected survival to a given age. The number of female elephants used to calculate the demographic series rose steadily, from 998 individuals in 1960 to 3802 individuals in 2018 (Fig. 3b; Table 2). Though not used directly in the PrR equation, changes in the maximum recorded lifespan and mother age at birth can indicate whether the period of study is sufficiently capturing representative demographic rates. Both variables were low at the start of the study period, in part because of the restrictive data selection criteria we imposed, and continued to rise well into the 2000s (Fig. 3c and d; Table 2). Currently, PrR is relatively stable: the oldest recorded age has remained 76 for the past 5 years, whilst there has been no increase in the oldest age at birth since 2011.

More importantly for the calculation of PrR, ages *B* and *M* (ages at 5 and 95% population fecundity respectively) have changed considerably with the length of the longitudinal data collection (Fig. 3e and f; Table 2). Age *B* reached 17 years in 1984, and has remained the same since then. However, the expected lifespan of females surviving to age B continued to increase up to 2015. For age *M*, there was a large increase, from 22 years in 1960 to 55 years in 2018. Unlike with the other demographic variables, age *M* has decreased for short periods, suggesting that it is far more susceptible to whether fertility rates are representative. It has, however, been fairly stable since 1998 (between 54 and 56 years, aside from a brief decline to 53 years in 2006 and 2007), and has remained unchanged in the last 4 years. Note that even though *e*_*M*_ has not increased much (Table 2), it is relative to the value of age *M*: elephants reaching age *M* are still expected to live for a number of years.

## Discussion

We find Asian elephants to have a statistically significant extended post-reproductive lifespan. Whilst the current population of Asian elephants is not fully wild, the elephants have better survival and later reproduction than zoo elephants [20, 21], and a comparable reproductive lifespan to wild elephants [19]. This could, therefore, be considered broadly representative of the species. Importantly, though the presence of a significant post-reproductive representation implicitly indicates early physiological reproductive cessation, this may not be the case. There is no clear cut age in Asian elephants after which further reproduction is impossible, unlike in e.g. humans [16] or killer whales [35]. Instead, fertility may be greatly reduced at old ages but still greater than zero [16]. As such, Asian elephants may be the first species identified as having an extended post-reproductive lifespan without an early physiological cessation of reproduction.

Physiological indicators of reproduction, such as ovarian activity, can definitively show whether individuals are incapable of further reproduction. One such measure was tested for toothed whales by Ellis et al. [14], and it would be interesting for future studies to investigate this in the Asian elephant (to our knowledge, no such data have been collected). Similarly, hormonal analysis could be one future direction for establishing whether there is physiological reproductive cessation (see e.g. zoo elephants, which are known to have severe reproductive problems [40, 41] and to be often acyclical/show changes in hormonal levels [42]). Though such physiological approaches to investigating post-reproductive lifespan can provide a better indication of reproductive cessation than PrR, they can be more challenging. Measuring ovarian activity following Ellis et al. [14] would require i) opportunistic sampling of dead individuals, ii) reliable ageing of these dead individuals, which requires the validation of age estimation techniques, and iii) there to be a non-linear relationship between corpora counts and age. Hormonal analysis, meanwhile, requires longitudinal study, and can be difficult to perform on wild, living individuals. At present, we cannot assess here whether cessation of reproduction is true physiological incapability of further reproduction or whether the lack of further reproduction is due to behaviour or declining body condition.

The opportunity to reproduce may often be out of an individual’s control, such as if dominant individuals suppress reproduction in subordinates [43–45]. Grandmothering may be a way for older individuals to gain fitness once the reproductive behaviours and mating preferences of others prevent further mating opportunities, or if a declining body condition at older ages affects fertility. Superseded reproductive females may switch to helping strategies, as seen in Seychelles warbler [46], carpenter bees *Xylocopa pubescens* [47], and social aphids *Quadrartus yoshinomiyai* [48, 49], without the need for early physiological reproductive cessation to evolve first (though *Q. yoshinomiyai* may actually have an early physiological end to reproduction [48]). However, as there is no evidence of dominance status being lost with age in the Asian elephant, this may not apply for this species. Regardless, post-reproductive lifespan is not inherently limited to species with early physiological reproductive cessation, but would still require some consistency in behaviour or somatic decline at the population level that decreases reproductive opportunities for females at older ages. Whilst we are currently unable to demonstrate ‘social menopause’, the findings here do not eliminate it as a possibility. Although the use of inter-birth intervals is problematic [13], our results show that older female elephants are generally not reproducing, and have not done so for many years.

Our results also highlight an important issue in assessing PrR for long-lived species: the need for sufficiently long follow-up in longitudinal datasets. Here, we find a PrR of 0.162, higher than the value calculated in a previous assessment with the same population (0.128) [16], indicating that values can change for species as long-term studies continue. Indeed, our analyses show that the point at which a longitudinally-studied species is assessed is crucial. Our selection criteria for elephants in early years led to elevated values for PrR, creating the striking contrast with the low values of the 1990s. A population with a more representative age structure at the beginning would still show similar extremes, though not necessarily following the same pattern, until age-specific fertility rates could be reliably calculated.

It is therefore important for future studies to consider when a species is assessed if they are calculating post-reproductive lifespan for long-lived species in which the study period is shorter than the typical adult lifespan. This issue can be alleviated by highly accurate age estimation for individuals of all ages, though there can be difficulties with verifying such estimation methods. For example, size cannot be used for accurate age estimation if growth is determinate and individuals have reached the asymptote of growth [30, 50]. Furthermore, as we find a difference between captive-born only and the captive-born and wild-caught samples, it is clear that PrR values are sensitive to the population of study, and to its present population dynamics [23]. For example, humans are widely regarded as menopausal, but PrR values can vary greatly between populations [13]. Whilst we do not dispute the post-reproductive status of those toothed whale species shown to be post-reproductive [3, 14], we wish to highlight that it is possible that other long-lived species have extended post-reproductive lifespan but currently lack sufficient data for statistical assessment.

## Conclusions

Due to the rarity of early reproductive cessation, we still do not know whether its evolution requires a specific driver or drivers. It may be that the prolonged post-reproductive lifespans of Asian elephants are currently driven by behaviour or body condition, rather than reproductive physiology, and may therefore be in an evolutionary transition; an extended post-reproductive life may be a prerequisite of an early end to reproductive capabilities. To properly tackle the puzzle provided by the evolution of early reproductive cessation and post-reproductive lifespan, we first need to know the taxonomic prevalence of these traits, underscoring the importance of long-term studies on known-age individuals.

## Supplementary information

**Additional file 1.** Dataset containing the *l*_*x*_ and *m*_*x*_ series of the population.

**Additional file 2.** Dataset containing the changing *l*_*x*_ and *m*_*x*_ series of the population (1960-2018).

## Data Availability

The R code used in this study is from the published literature, and slightly modified as outlined in the Methods. The *l*_*x*_ and *m*_*x*_ series for the elephants can be found as Supporting Information files (Additional files 1 and 2).

## Abbreviations

PrR: Post-reproductive representation
